# Can cognitive insight predict symptom remission in a first episode psychosis cohort?

**DOI:** 10.1186/s12888-017-1210-9

**Published:** 2017-02-06

**Authors:** Jennifer A. O’Connor, Lyn Ellett, Olesya Ajnakina, Tabea Schoeler, Anna Kollliakou, Antonella Trotta, Benjamin D. Wiffen, Aurora M. Falcone, Marta Di Forti, Robin M. Murray, Sagnik Bhattacharyya, Anthony S. David

**Affiliations:** 1 Department of Psychosis Studies, P068, Institute of Psychiatry, Psychology and Neuroscience, De Crespigny Park, London, SE5 8AF UK; 2 Department of Psychology, Royal Holloway, University of London, Egham, Surrey, TW20 0EX UK; 3 Department of Psychological Medicine, Institute of Psychiatry, Psychology and Neuroscience, De Crespigny Park, London, SE5 8AF UK

## Abstract

**Background:**

The outcome of first episode psychosis (FEP) is highly variable and difficult to predict. Cognitive insight measured at illness onset has previously been found to predict psychopathology 12-months later. The aims of this study were to examine whether the prospective relationship between cognitive insight and symptom severity is evident at four-years following FEP and to examine some psychological correlates of cognitive insight.

**Methods:**

FEP participants (*n* = 90) completed the Beck Cognitive Insight Scale (BCIS) at illness onset, and associations between BCIS scores with symptom severity outcomes (4-years after FEP) were assessed. The BCIS scales (*self-reflectiveness* and *self-certainty*) were examined as a composite score, and individually compared to other cognitive measures (IQ and jumping to conclusions (JTC) bias).

**Results:**

Regression analyses revealed that the cognitive insight composite did not predict 4-year symptom remission in this study while the self-reflection subscale of the BCIS predicted severity of symptoms at 4-years. Self-certainty items of the BCIS were not associated with symptom severity. Significant correlations between the JTC bias, self-certainty and IQ were found, but self-reflection did not correlate with these other cognitive measures.

**Conclusions:**

Self-reflective capacity is a more relevant and independent cognitive construct than self-certainty for predicting prospective symptom severity in psychosis. Improving self-reflection may be a useful target for early intervention research.

## Background

The study of higher-order thinking in psychosis populations has been examined using various cognitive constructs. Self-reflective capacity is a meta-cognitive construct defined as the ability to be accurately introspective, and recognise one’s own subjective fallibility [1]. It has been suggested that impaired self-reflection may lead to poor sensory and narrative integration of experience, increasing the risk of psychiatric symptoms [2]. A different but related construct relevant to psychosis research is ‘over-confidence in judgement’, which is thought to manifest behaviourally in reasoning biases such as an early acceptance of incorrect ideas and failure to consider alternatives, and is often found associated with delusional beliefs [3–5]. Beck and colleagues suggest that self-reflection and confidence in judgement are related though psychometrically distinct concepts, such that a high level of self-certainty might diminish one’s ability or willingness to be introspective [6]. Likewise a sound self-reflective capacity may enable one to redress reasoning biases [6].

The Beck Cognitive Insight Scale (BCIS) [7] examines these two theoretically driven and empirically derived factors: self-certainty, which assesses over-confidence and certainty about being right (e.g. *‘I know better than anyone else what my problems are’*), and self-reflectiveness, which assesses willingness to accept fallibility and external feedback, as well as recognising dysfunctional reasoning style (e.g. *‘Some of the ideas I was certain were true turned out to be false’*). A composite index can be calculated by subtracting self-certainty from self-reflective scores. Thus high self-reflectiveness and low self-certainty is the formula for good ‘cognitive insight’ [7].

It is proposed in this study, that limited cognitive insight increases one’s risk for experiencing enduring psychotic symptoms over time. Earlier analyses from our research group found that cognitive insight at first-episode psychosis (FEP) is a predictor of overall symptom severity at 12-month follow-up [8]. This finding supports other research which found that cognitive insight is cross-sectionally associated with symptom severity across a range of positive symptoms in psychosis [9–12]. Until now, the question of whether cognitive insight prospectively predicts symptom outcome beyond 12 months has not been examined.

Understanding the individual BCIS scales within a broader neuro-cognitive framework is also important, and the question of *how* the individual subscales underlying cognitive insight (self-reflection and self-certainty) operate in psychosis populations still requires scrutiny. While the general consensus is that individuals with psychosis show lower-levels of self-reflection and greater-levels of self-certainty [9, 11, 13, 14] there are studies that report contradictory findings, with some failing to show a significant difference on BCIS subscale scores between people with schizophrenia and healthy controls [15, 16]. Furthermore, Köther et al. [17] demonstrated that self-certainty ratings were actually lower for people with schizophrenia. Elucidating the patterns of association that the BCIS scales have with other cognitive variables might help to explain how cognitive insight is relevant to clinical outcome in psychosis.

While our research group previously found cognitive insight to be distinct in its predictive value from other neuropsychological variables such as executive function, IQ and clinical insight (i.e. one’s awareness of their own psychosis) [18, 19] we did not explore how the BCIS measure relates to other ‘reasoning bias’ type cognitive constructs which are often examined in psychosis research. One such construct is the ‘jumping to conclusions (JTC)’ data-gathering bias [20]. Whilst the relationship between JTC and psychosis is well-established [4, 21, 22]_,_ it is still unclear whether JTC behaviour is an accurate measure of ‘overconfidence in judgement’ or measures nothing more than hasty decision making [23]. For this reason, understanding how this data gathering bias correlates with a *direct* measure of over-confidence (i.e. BCIS self-certainty) is of theoretical interest and may help broaden our knowledge of how self-certainty operates in FEP. It is also important to consider IQ when studying the correlates of these higher-order constructs given the relevance of neuropsychological aspects of cognition to prognosis in psychosis [24, 25].

In light of the current gaps in the literature as described, the aim of the current study was to examine whether cognitive insight can predict four year symptom severity in an FEP sample previously described [8] and explore how the underlying components of cognitive insight (self-certainty and self-reflection) are associated with other cognitive factors (IQ and JTC).

## Methods

### Participants

First episode psychosis patients (*n* = 111) were recruited as part of the National Institute of Health Research (NIHR) and Biomedical Research Centre (BRC), Genetics and Psychosis study. Selection criteria required participants to be aged 18–65 years, who met DSM-IV criteria for psychosis, and presented to the selected boroughs in South London adult mental health services (identified through examination of the clinical notes of new psychiatric admissions and consultation with clinical teams). Further inclusion criteria were applied: contact with psychiatric services for psychosis ≤6 months; fluent English speaker; psychosis identified as having a non-organic cause (e.g. differential diagnoses such as medically induced psychosis i.e. deliriums, a history of head injury or neurological condition were exclusions). All those patients identified as eligible, were approached as soon as possible and invited to take part in the study. Research diagnoses were provided by qualified psychiatrists subject to inter-rater reliability checks (Intra class correlation = .97) using the Operational Criteria for studies of psychotic illness (OPCRIT) [26].

### Measures

Demographic data were collected from self-report, supplemented by clinical records, at study entry.

#### Cognitive measures

Cognitive insight was measured using the Beck Cognitive Insight Scale: BCIS [7]. This is a 15 item self-report scale, with items rated from ‘*do not agree at all’* to ‘*agree completely’*. There are two subscales which measure participant endorsement of: ‘self-reflectiveness’ (nine items, range 9 to 63), and ‘self-certainty’ (six items, range 6 to 42). A cognitive insight score was derived by deducting the BCIS self-certainty scale item total from the BCIS self-reflective scale item total. Consistent with our previous research [8], each BCIS item was rated on 7- point scale instead of the original 4-point scale, to increase the precision of this measure [27]. Confirmatory factor analysis conducted in this study sample suggests that factor loadings for both constructs were equivalent to the original item loadings [7]*,* and the two factor model reached statistical significance (t-ratio >1.96) [28]. Cronbach’s α for the self-certainty and self-reflective scale in this study was 0.76 and 0.71 respectively, higher than the consistencies achieved in the original BCIS publication paper [7].

The ‘Jumping to Conclusions’ or JTC bias, was measured by the behavioural response to a probabilistic reasoning paradigm called ‘The Beads Task’ [29]; in the version used for this study, participants were shown two jars that contain coloured beads (orange or black). Each jar contained beads in a different proportion, e.g. one jar contains 85 black and 15 orange beads, and the other jar contains the reverse proportion. Participants were informed of and shown the coloured bead proportions, before the containers were removed from view. Participants were then told that each jar (either the jar containing mainly orange beads or that containing mainly black beads) has the same probability of being chosen by the researcher (50:50) and that beads will be extracted from the selected jar and shown to participants one at a time. It was the participant’s task to decide from which of the two jars the beads were being taken; the mainly orange or the mainly black jar. They were told that they should only decide when they are certain. We adopted the ‘two or less draw to decision threshold’ measure to identify JTC bias, as this measure has been shown to be most reliably associated with delusions [30, 31]. A tendency to ‘Jump to Conclusions’ was operationally defined as the respondent making a decision after two beads or fewer, as this threshold has been used in other FEP studies [32, 33]. To estimate IQ, a short version of the Wechsler Adult Intelligence Scale -Third Edition: WAIS III [34] was administered and included the following subtests: Information, Digit Span, Block Design, Matrix Reasoning and Digit Symbol Coding. These particular subtests were chosen because they index a wide range of cognitive abilities, including all relevant IQ domains. These scores were averaged within their domain and multiplied by the total number of WAIS III subtests in each domain to approximate an individual IQ score; using short-forms of WAIS is common in psychosis research to estimate full scale IQ [35–38].

#### Psychopathology

The Positive and Negative Syndrome Scale (PANSS) [39] was used to rate symptoms at study entry. Only the positive and negative scales were examined in this study. Each item is scored on a scale of 1 to 7: absent, minimal, mild, moderate, moderate severe, severe and extreme. A score of 4 (moderate) or higher indicates the presence of clinical psychopathology. Item ratings were completed through interview with participants and by collecting collateral information from healthcare workers based on 7 days prior to assessment. Inter-rater agreement coefficients for rating pairs (*n* = 22) were calculated using a Spearman-Brown formula (agreement amongst multiple observers corrected for number of observers). Mean level of agreement was *r* = 0.814, which is above conventionally accepted thresholds for adequate inter-rater agreement [40].

The Global Assessment of Functioning (GAF) [41] was used to measure overall illness severity at study entry and at follow-up. The GAF is a widely used observer-rated instrument to determine clinical and functional status on a scale from 1 to 100. We measured symptom experience (GAF-S) separately to functional disability (GAF-F). Making this distinction between these two recovery types on the GAF has been shown to improve the psychometric properties of this measure [42]. Information for GAF-S ratings were derived from “any source, such as direct interview of the patient, a reliable informant, or a case record” [41] (p767)_._ In this study, clinical notes were used or when possible, GAF-S was rated following face to face interview. Raters were subject to inter-rater reliability checks, achieving excellent intra-class correlations when rating GAF-S from clinical records (ICC > 0.90) [43]. Further, GAF-S scores collected from clinical records compared to GAF-S scored via face to face interview showed high comparability (ICC = .81) [43].

### Design and procedure

**Fig. 1:**
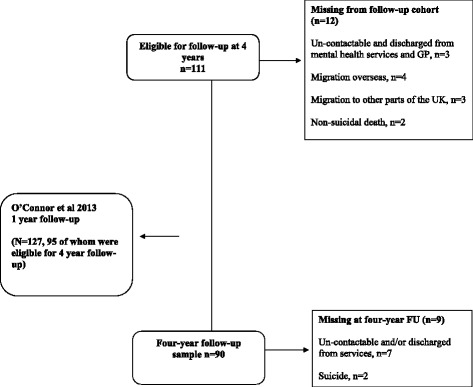
Flowchart indicating follow-up methodology and attrition rate

The study used a within subject longitudinal design to analyse relationships between cognitive insight collected at the first onset of psychosis and GAF-S collected at 4-year follow-up. The initial sample of interest (*n* = 111) consisted only of participants who completed all cognitive measures of interest to this study at baseline (87% of total sample evaluated at O’Connor et al. 12 month follow-up study [8]). Baseline variables (GAF-S, JTC, IQ, PANSS, demographic factors and diagnoses) were analysed as possible covariates, and to characterise the patient sample. Participants completed measures at follow-up with an attrition rate of 18.9% (see Fig. 1 for details). The majority of this sample (95%) formed part of the 12 month follow-up in our previous study [8]. The average follow-up time was 49 months, or just over four years post FEP (sd = 11.5, range 27–86 months).

### Data analysis

Hierarchical multiple regression was conducted with GAF symptoms at four-years as the dependent variable and cognitive insight (BCIS composite score) as the predictor variable. Analyses of Pearson’s and point bi-serial correlations were conducted to examine relationships between covariates and the dependent variable (GAF-S at 4 year follow-up); covariates which correlated highly with the outcome measure (*p* < .01 *and* of greater magnitude than *r* = .25) were entered into subsequent regression modelling [44], before cognitive insight was entered at step 2. Correlation analyses enabled the investigation of relationships between the BCIS scales and other cognitive variables (IQ and JTC bias).

## Results

### Descriptive baseline data

Table 1 describes the demographic, clinical and cognitive characteristics of this sample that completed follow-up at four-years after FEP (*n* = 90). Participants were classified into eight different diagnostic categories according to the DSM IV. The most common diagnosis was schizophreniform disorder (28.8%) and the majority of participants (57.7%) were classified as having a non-affective psychosis. In terms of symptom severity, the mean PANSS positive and negative symptom subscale scores suggest that the average participant was ‘mildly unwell’ at baseline assessment. In terms of cognitive descriptive data, other than those reported in Table 1, the self-reflective scale (SR) and the self-certainty scale (SC) scales were negatively correlated (*r* = −.253). The mean IQ score was in the low-average range commensurate with previous FEP findings [45]. Nearly half the sample (45%) showed a JTC bias according to a <2 beads threshold, which is also consistent with previous FEP findings [32].

**Table 1:** Sample Characteristics (*N* = 90)

Demographic characteristics	
Age, years:	Mean (sd)
	29 (9.1)
Gender	62% male
Ethnicity (n)	
White British	24
Black African	23
Black Caribbean	15
White European	8
Mixed Race	9
Asian	7
Other	4
Clinical Characteristics	
Diagnosis (n)	
Schizophreniform disorder	26
Manic episode with psychosis	16
Schizophrenia	13
Psychosis not otherwise specified	12
Major depression with psychotic features	10
Schizoaffective disorder depressed	8
Schizoaffective disorder bi-polar	4
Delusional disorder	1
PANSS	Mean (sd)
Positive symptoms	14 (5.8)
Negative symptoms	15 (6.4)
Cognitive characteristics	
BCIS	Mean (sd)
Composite Index	14.33 (14.05)
Self-reflection	38.40 (9.72)
Self-certainty	24.07 (7.97)
IQ: Mean (sd)	90.39 (15.35)
JTC^a^	4.94 (5.30)

^a^Descriptive data represents draws to decisions on the Beads Task

### Follow-up data

There were no significant differences between those who were traceable for 4-year follow-up assessment (*n* = 90) versus those who had become untraceable at follow-up (*n* = 21), with the exception of baseline GAF-S scores, in that those who were untraceable at follow-up had significantly more psychopathology at baseline: *t* (109) = −2.341, *p* = 0.012. Compared to baseline, GAF symptom severity was significantly lower at 4-year follow-up (*t* (87) = −4.816, *p <* 0.001 respectively). A 4-years, the cohort mean GAF symptom score was above remission thresholds for FEP (>59) [46].

### Main analysis

Correlation analysis was conducted to determine which baseline predictors should be entered into predictor models. Only variables that correlated significantly with GAF symptom outcome at 4 year follow-up as outlined above were entered into subsequent regression models. The GAF Time-0 symptom measure did not correlate with GAF symptoms as 4 year follow-up. GAF symptom scores at four-year follow-up were significantly correlated with baseline negative symptoms (*r* = −.320) and diagnosis (r_pb_ = .294) such that less severe negative symptoms and an affective diagnosis at FEP onset was associated with decreased psychopathology at 4-year follow-up. Therefore these variables were entered into the hierarchical regression at step 1, and cognitive insight was entered at step 2. Overall, the model was significant and negative symptoms, diagnosis and cognitive insight accounted for approximately 15% of variance in symptom outcome [F(3,79) = 4.797, p = 0.002]. However, cognitive insight uniquely accounted for just 0.6% of variance in symptom outcome, which was not a significant individual contribution to the model [F (3, 79) = .585 p = 0.447 R^2^ = .148, adjusted R^2^ = .115)].

Post hoc analysis of the BCIS scales individually revealed that self-reflectiveness was significantly correlated with symptom outcome at 4-years such that greater ability to self-reflect was associated with less severe symptoms prospectively (*r* = .25), although not cross-sectionally, at baseline (*r* = −.09). Conversely, self-certainty did not correlate with symptom outcome at 4-years (*r* = .021) although its relationship with symptoms cross-sectionally at baseline was nearing significance (*r* = −.18, *p* = 0.08).

The two BCIS subscales were entered separately in the regression model to identify unique contributions of self-reflectivity and self-certainty on symptom outcome at 4 years. Overall, the model was significant (*F* (4, 78) =5.258, *p* = 0.046), and by entering the BCIS subscales separately, an increase in 6.5% of variance in symptom outcome was explained (adjusted R^2^ = .172). Higher scores on the self-reflectiveness sub-scale predicted significantly higher GAF-S scores (i.e. less severe psychopathology) at Time 2 follow-up (*t* (78) =2.324, *p =* .023). The self-certainty sub-scale did not contribute significantly to the variance (*t* (78) =1.57, *p =* .199). Table 2 reports the unique contribution of each variable entered into the regression model.

**Table 2:** Hierarchical regression to predict symptom severity at four-year follow up

	Std β	T	p
Time 0 negative symptoms	−0.247	−2.356	0.021
Diagnoses ^a^	0.247	2.378	0.020
BCIS self-reflective scale	0.245	2.324	0.023
BCIS self-certainty scale	0.167	1.577	0.119

^a^affective vs. non-affective psychosis

In summary, analyses of the BCIS scales individually revealed that self-reflectiveness was significantly correlated with symptom outcome *prospectively*. The self-certainty subscale was not associated with later symptom outcome, although its relationship with symptoms cross-sectionally at baseline approached significance (higher self-certainty related to greater concurrent symptom severity).

Finally, in terms of the BCIS subscales’ relationship to other cognitive variables, the self-certainty items had a significant, though weak association with the JTC bias (*r* = −.258) and IQ (*r* = −.313) such that higher self-certainty was associated with a tendency to jump to conclusions, and lower IQ. Neither the JTC measure, nor IQ was significantly correlated with the BCIS self-reflective scale (*r* = .029, and *r* = .033 respectively).

## Discussion

Cognitive insight (as measured by the BCIS composite score) did not predict symptom severity at four years post FEP, but the self-reflective sub-scale did. Participants who endorsed highly self-reflective behaviour, i.e. greater agreement with scale items such as, “*some of my experiences that have seemed very real may have been due to my imagination”,* had fewer and less severe psychopathological symptoms at four-years after psychosis onset, compared to those individuals who did not endorse these items. Direct and indirect measures relating to confidence in judgement (BCIS self-certainty scale and JTC bias) did not contribute to symptom outcome, though these factors correlated significantly with each other, and IQ.

These findings contradict some previous studies, which found that the correlation between the combined cognitive insight construct and symptom outcome is stronger than that between individual subscales and symptom outcome [8, 9, 47]. However, previous studies have only examined the cross-sectional and short-term prospective relationships between the cognitive insight and symptom outcome and this is the first study to examine these relationships in the medium-term (four years) after FEP. This suggests a complex longitudinal relationship between the BCIS and clinical status, such that aspects of the BCIS measuring meta-cognitive thinking (self-reflective items) have a more *prospective* relationship to symptom experience, whereas those items indexing ‘confidence in judgement’ (self-certainty items) tend to correlate more highly to concurrent symptoms.

The converging associations between JTC, self-certainty and IQ support a growing body of evidence that reasoning processes are underpinned by general intellectual functions [48, 49]. For instance, the literature suggests that schizophrenia and delusional prone participants tend to show ‘overconfident judgement’ in relation to their own objective ‘errors’, but they do not show the same level of conviction for their correct responses on tasks (see Balzan et al. for a review [23]). The association found between the JTC and BCIS self-certainty scale is also relevant to contention in the field as to whether the JTC beads task is measuring more than hasty decision making [23]. Our findings tentatively support the notion that the JTC Beads task is perhaps tapping into the same ‘confidence in judgement’ factor as the BCIS self-certainty scale.

Our, results also suggest that self-reflective aspects of thinking are unrelated to other measured cognitive constructs (JTC or IQ) consistent with reports from recent meta-analyses, that the BCIS self-reflective scale had fewer neuropsychological correlates than the self-certainty scale [49]_._ By virtue of its lack of correlation with neuropsychological function, poor self-reflective capacity may be more amenable to change through psychological support. Indeed, psycho-social interventions have been shown to change participant endorsement of the self-reflective scale items, but not self-certainty items [9, 50, 51].

It would be valuable to know whether self-reflection can predict the severity of some specific psychotic symptoms more than others (i.e. hallucinations vs. delusions). Understanding this would be particularly informative, given recent calls for the development of symptom-specific interventions in psychosis [52]. Indeed one previous CBT for psychosis intervention found that higher endorsement of the BCIS self-reflection items was associated with clinically significant delusional improvements, but was *not* linked to changes in hallucinations [47].

Our results should be considered in view of the limitations of the study. Follow-up assessment occurred when participants were willing to be interviewed, or when clinical records were available, and so did not always occur strictly within 4-year window. In terms of generalisability, those participants missing at follow-up did have significantly more severe psychopathology at baseline on the GAF symptom measure than those participants retained at follow-up. This may have caused some sample bias, but its effect on the strength of association with BCIS measures is unclear. Evidence suggests that acuteness of psychosis at illness-onset predicts *better* symptom outcomes [53], which means this study might be underestimating the rates of positive recovery outcomes [54]. Nevertheless, it seems unlikely that this identified difference between those lost at follow-up and the follow-up sample, impacted upon the main findings, given that the baseline GAF symptom severity was not a confounding variable in the regression model. Finally, it is unfortunate that data on BCIS and JTC were not collected at follow-up as it would have been useful to more directly measure the stability of these constructs over time.

## Conclusion

Through employing a longitudinal design, we present evidence that subjective self-reflection has an important role in predicting symptom remission in early psychosis. These findings reveal a potentially important target for psychological intervention.
